# Facial emotion recognition is associated with executive functions and depression scores, but not staging of dementia, in mild‐to‐moderate Alzheimer's disease

**DOI:** 10.1002/brb3.3390

**Published:** 2024-01-24

**Authors:** İsmail Buçgün, Şükrü Alperen Korkmaz, Demet Güleç Öyekçin

**Affiliations:** ^1^ Private Practice Adana Turkey; ^2^ Department of Psychiatry Faculty of Medicine Çanakkale Onsekiz Mart University Çanakkale Turkey

**Keywords:** Alzheimer's disease, dementia, facial emotion recognition, facial expressions, social cognition, stage of dementia

## Abstract

**Background:**

Although deficits in facial emotion recognition (FER) significantly affect interpersonal communication and social functioning, there is no consensus on how FER affects Alzheimer's disease (AD). In this study, we aimed to investigate the clinical and neuropsychological factors affecting the possible deficits in the FER abilities of patients with AD.

**Methods:**

This cross‐sectional study included 37 patients with mild [clinical dementia rating (CDR) scale score = 1] or moderate (CDR = 2) AD, in whom vascular dementia and depression were excluded, and 24 cognitively normal (CDR = 0) subjects. FER ability was determined using the facial emotion identification test (FEIT) and facial emotion discrimination test (FEDT). All participants underwent mini‐mental state examination (MMSE), frontal assessment battery (FAB), and geriatric depression scale (GDS). The neuropsychiatric inventory‐clinician rating scale (NPI‐C), Katz index of independence in activities of daily living, and Lawton instrumental activities of daily living were also administered to patients with AD.

**Results:**

The FEIT and FEDT total scores showed that patients with mild and moderate AD had significant FER deficits compared to healthy controls. However, no significant difference was observed between patients with mild and moderate AD in the FEIT and FEDT total scores. FEIT and FEDT scores were not correlated with the MMSE and NPI‐C total and subscales scores in patients with AD. Linear regression indicated that FEIT and FEDT total scores were significantly related to age and FAB scores. The GDS score negatively moderated the relationship between FAB and FEDT.

**Conclusions:**

This study demonstrated a decreased FER ability in patients with AD. The critical point in FER deficits is the presence of dementia, not the dementia stage, in AD. It has been determined that executive functions and depression (even at a subsyndromal level), which have limited knowledge, are associated with FER abilities.

## Graphical abstract

Facial emotion recognition (identification and discrimination of facial emotions) was impaired in patients with mild and moderate Alzheimer's disease compared to healthy controls, whereas there was no difference among those with dementia.

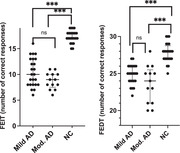

## 1 INTRODUCTION

Alzheimer's disease (AD) is the most common type of dementia that leads to impairments in memory and learning and many cognitive dysfunctions, such as executive functions, complex attention, visuospatial abilities, and language (Diagnostic and statistical manual of mental disorders, fifth edition [DSM‐5]). In addition to these core clinical symptoms, social cognitive changes impair facial emotion recognition (FER), an important domain affected in patients with AD (McCade et al., 2011; Takahashi et al., 2020). FER, which modulates social behaviors and is an important dimension of interpersonal relationships, involves the recognition of emotions through facial expressions (Henry et al., 2016). Recognizing facial expressions of emotions provides information about the emotional states of other individuals and predicts probable actions, thus enabling individuals to adapt to the environment and participate in social activities, social interactions, social bonds, and meaningful relationships, which are integral parts of a healthy life (García‐Casal et al., 2017; Huber et al., 2011). Consequently, emotion recognition impairment can significantly impact interpersonal functioning and is associated with depression, inappropriate social behavior, and psychobehavioral disturbance commonly seen in dementias (Chiu et al., 2006; Phillips et al., 2010; Shimokawa et al., 2001). These issues increase caregiver burden and make appropriate treatment and care more difficult to achieve (Shimokawa et al., 2003; Takahashi et al., 2020).

When studies of emotion recognition from facial expressions in AD are examined, conflicting results have shown that this ability is impaired (Kohler et al., 2005; Phillips et al., 2010; Roudier et al., 1998) and preserved (Freedman et al., 2013; Hsieh et al., 2012; Lavenu et al., 1999). Furthermore, in some studies, difficulty in facial expression recognition in patients with AD was associated with global cognitive status (Bertoux et al., 2015; Torres et al., 2015), but not in others (Cosentino et al., 2014; McLellan et al., 2008). In addition, it is unclear whether there is a relationship between executive functions and FER. Neuropsychiatric symptoms such as apathy, aggression, disinhibition, and irritability are known to affect social cognition and social functioning (Lyketsos et al., 2011). Facial recognition of emotion, integrated with other social information, allows the observer to make assumptions about what an individual is thinking or feeling and responds to their needs accordingly. Therefore, an inability to read the emotional states of others can lead to patients with AD being perceived as withdrawn or socially inappropriate (Adolphs, 2009). This may mean that patients with AD with FER deficits may have difficulty maintaining activities of daily living. These problems may be associated with an increase in the burden of care and may be a potentially critical factor in reducing the quality of life (McLellan et al., 2008).

Few studies have compared the facial recognition abilities of patients with mild cognitive impairment (MCI) and AD at different stages (Dourado et al., 2019; Lavenu & Pasquier, 2005; Sheardova et al., 2014; Spoletini et al., 2008; Weiss et al., 2008). Although there is some evidence that impairment in recognizing emotions increases with progression in the AD stage (Adolphs, 2009; Lyketsos et al., 2011; Spoletini et al., 2008), studies have shown that it is preserved in severe stages of the disease (Sheardova et al., 2014), indicating that this issue needs to be clarified.

This study was designed because of the inconsistent and uncertain results mentioned above. In this study, we aimed to (I) investigate whether there is a difference between patients with mild and moderate AD and healthy controls in terms of FER, (II) examine the relationship between disease severity and FER ability in AD, and determine which sociodemographic and clinical factors such as global cognitive and executive functions, neuropsychiatric, and mood symptoms are associated with impaired ability according to disease severity. Understanding the nature and extent of FER deficits in AD and their relationship with clinical severity, cognitive function, neuropsychiatric symptoms, and activities of daily living may provide new insights into the social and behavioral impairments that often accompany this disease.

## 2 METHODS

### 2.1 Participants

In this cross‐sectional study, we recruited 37 participants diagnosed with possible or probable mild and moderate AD and 24 cognitively normal (CN) subjects. Patients with AD who underwent medical examination between May 2019 and May 2020 were recruited from the outpatient clinic of the Department of Psychiatry, Çanakkale Onsekiz Mart University Hospital, Turkey. The diagnosis of AD was confirmed using the DSM‐5. CNs were recruited through an announcement posted at Çanakkale Onsekiz Mart University and by word of mouth. Age–sex‐ and education‐level matched CN subjects included based on the following criteria: no memory complaints, no history of psychiatric or neurological disorders, no history of dementia diagnosis, mini‐mental state examination (MMSE) score ≥26, geriatric depression scale (GDS) score <14, and clinical dementia rating (CDR) scale score = 0 (no dementia).

Demographic characteristics (age, sex, occupation, marital status, and education level), clinical information (duration of AD, age at onset of AD, current psychical diseases, and use of regular drugs), mental disorders and dementia in first‐degree relatives, cigarette consumption, and body mass index were recorded. All participants underwent routine eye examination for visual deficits. Patients were excluded if they had a diagnosis of dementia other than AD, concurrent psychiatric diagnosis, the presence of neurological disorders affecting the central nervous system, history of head trauma, stroke, epilepsy, alcohol or substance abuse, profound visual or hearing deficit, and use of drugs causing cognitive impairment, such as benzodiazepines. None of the AD patients had prosopagnosia. In addition, patients with a Hachinski ischemic scale score >4 (Hachinski et al., 1975) or global CDR scale score >3 (Morris, 1993) or patients and CNs with a GDS score >14 (Yesavage, 1988) were not included the study. All participants were at least 60 years old, had primary school education, and lived in their homes in the community. Interviews were also conducted with the caregivers accompanying the patients.

The study was conducted in accordance with the Declaration of Helsinki and approved by the University Ethics Committee. All patients and/or their caregivers and CNs provided informed consent to participate in this study.

### 2.2 Neuropsychological evaluation

The MMSE, CDR scale, and frontal assessment battery (FAB) were administered to all participants to measure their global cognitive, staging of dementia, and executive functions, respectively (Folstein et al., 1975; Hurtado‐Pomares et al., 2018). Although none of the participants had a major depressive disorder, the GDS was used to determine the subthreshold depression symptoms (Yesavage, 1988). The CDR was used to measure disease severity in patients, and scores of 0, 0.5, 1, and 2 indicated no dementia, MCI, and mild and moderate AD, respectively (Morris, 1993). The CDR‐sum of boxes (CDR‐SB) is also used as a functional impairment scale, scored on a scale of 0–18 by summing the box scores (higher scores indicate more impairment). The neuropsychiatric inventory‐clinician rating scale (NPI‐C), Katz index of independence in activities of daily living (Katz ADL), and Lawton instrumental activities of daily living (IADL) were also administered to patients with AD.

We used the Turkish‐validated version of the MMSE, which includes a test of orientation, registration, short‐term memory, language, comprehension, and basic motor skills. The total score ranges from 0 to 30, with lower scores indicating impaired global cognitive function (Keskinoglu et al., 2009).

The FAB was used to evaluate frontal lobe function in patients with AD. It assesses frontal functions such as conceptualization, mental flexibility, programming, sensitivity to interference, inhibitory control, and peripheral autonomy. It has been reported to help assess executive function in clinical settings (Hurtado‐Pomares et al., 2018). A higher score indicated better performance.

The NPI assesses neuropsychiatric symptoms (Cummings et al., 1994). The NPI‐C used in this study was a revised version of the NPI (de Medeiros et al., 2010). In contrast to the NPI, the clinician synthesizes relevant information from the caregiver or family member, interviews the patient, and directly observes their behavior to generate the final ratings for each item in each domain of the NPI‐C. The NPI‐C scale allows for better interpretation and ratings of neuropsychiatric symptoms (Stella et al., 2015). Each of the 14 domains consists of a screening question and 6–16 subsequent questions about specific behaviors, which can be rated in terms of the frequency of occurrence and severity (Şahin Cankurtaran et al., 2015).

The Katz ADL scale assesses basic daily self‐care capacity and is generally used in community‐dwelling older adults (Katz, 1963). The six‐item Katz ADL is short and can be administered through an interview using a dichotomous rating. The IADL scale is appropriate for assessing independent living skills, such as telephone use, shopping, housekeeping, and laundry (Lawton & Brody, 1969). These skills are more complex than the basic activities of daily living. Eight domains of function were measured using the IADL scale (Isik et al., 2020). The summary score ranges from 0 (low function, dependent) to 8 (high function, independent).

### 2.3 Facial emotion recognition assessment

The ability to recognize facial emotional expressions was evaluated in both groups using the following two tasks: the facial emotion identification test (FEIT) and the facial emotion discrimination test (FEDT). The FEIT shows 19 black‐and‐white pictures of 6 basic emotions (happiness, sadness, anger, surprise, disgust, and shame) for the participant to identify. The subject was given a 19‐item answer key, with 6 basic emotions written as options for each question. The subjects were asked to mark which of the six basic emotions written on the key in their hand most closely matched the emotion in each photograph while watching the photographs in turn. A score of 1 was given for correct answers and 0 for incorrect answers. The highest score that can be obtained from the test is 19 (Erol et al., 2017; Kerr & Neale, 1993). Although few studies have evaluated this test in AD (Nedelska et al., 2010), it has been adequately studied in mental disorders such as schizophrenia, bipolar disorder, and alcohol and cannabis dependencies (Aşık & Ünsal, 2020; Bayrakçı et al., 2015; Işık Ulusoy et al., 2020; Lecomte et al., 2019; Li et al., 2010).

FEDTs, such as the FEIT, have been studied primarily for mental disorders. The FEDT consists of 30 pairs of black and white photographs, each showing two people displaying one or two of the six emotions depicted in the FEIT. The task of this test was to determine whether two people in each pair had the same or different emotions. As in FEIT, the pictures remained on the screen for 15 s each, with a 10‐s blank between each picture. The participant was given a 30‐item response form, each with 2 options: “same” or “different.” After each stimulus, the participant was asked to mark their answer on the answer form, and the score was calculated as the number of correct answers (0–30) (Isik et al., 2020; Kerr & Neale, 1993).

FEIT and FEDT started with a screen where the task instructions were presented to the participant in Turkish. Simultaneously, the examiner read these instructions aloud to the participant to ensure minimal variation in the practice procedure. Following the instructions, three practice stimuli that were not part of the test set were presented. The instruction and practice trials were repeated if the participant failed to understand the instruction. The answers are marked on the response form, and the participant is asked to indicate verbally which emotion he/she is thinking of, to ensure that the emotion guessed is the same as the emotion marked. The test was terminated if the participant still did not understand the test instructions or did not know how to respond after the instructions, and the test was repeated.

### 2.4 Statistics

Data were analyzed using the Statistical Package for Social Sciences (SPSS) version 28.0 (SPSS 28.0, IBM). The figure was drawn with GraphPad Prism 9.3.1 (GraphPad Software Inc.). Before analyzing the data, they were checked for loss and extreme values (outliers). No significant outliers were observed. The normality of data was assessed with the skewness and kurtosis (whether the values were between −1.5 and +1.5), Kolmogorov–Smirnov, and Shapiro–Wilk tests. Sociodemographic and clinical characteristics were summarized as frequencies and percentages for categorical variables and as mean ± standard deviation (SD) or median and minimum–maximum for continuous variables, as appropriate. Analysis of variance, independent samples *t*‐test, Mann–Whitney *U* test, and Kruskal–Wallis test were used to compare the groups, as appropriate. When the primary analysis reached statistical significance, post hoc analysis was performed for multiple comparisons using the Bonferroni test when variances were equally distributed and Dunnett's T3 test when variances were not equally distributed. The chi‐square test was used to compare categorical variables. Spearman's correlation analysis was used to assess the association of the FEIT and FEDT with 14 subscale scores on the NPI‐C scale, FAB, GDS, and MMSE.

Age, sex, educational level, and total duration of illness were included as covariates in the analyses to assess their effects on FEIT and FEDT. For this purpose, we used a nonparametric analysis of covariance equivalent test (Quade's ANCOVA). Factors that may predict the FEIT and FEDT scores were evaluated using regression analysis (enter method). The adjusted *R* ^2^ value was used to determine the variance of the dependent variables explained by the model. To examine the relationship between FEIT and FEDT with the variables, moderator analysis was conducted to determine whether any variables served as a moderator variable. The significance level was set at *p* = .05, and all tests were 2‐tailed.

## 3 RESULTS

### 3.1 Sociodemographic and clinical data

According to the CDR, 24 of the 37 patients were considered to have mild AD (CDR score = 1), and 13 of the 37 were in the moderate AD (CDR score = 2) groups. Furthermore, 24 CNs were included in the study. Table 1 summarizes the demographic and clinical characteristics of the study participants. There were no significant differences in age, sex, education level, marital status, or working status among the three groups (all *p* > .05). In addition, no significant differences were found in smoking, physical illness in the three groups, or psychotropic medication use in patients with AD (all *p* > .05).

**TABLE 1:** Demographic and clinical characteristics of the subjects.

Characteristics	Mild AD (CDR 1)	Moderate AD (CDR 2)	CN (CDR 0)	*p*
	(*n* = 24)	(*n* = 13)	(*n* = 24)	
Age (years, mean ± SD)	75.3 ± 6 Range = 64–85	77.4 ± 5 Range = 68–84	75.6 ± 6 Range = 64–85	.521
Gender (female)	19 (79.2%)	6 (46.2%)	11 (45.8%)	.06
Disease duration (years, mean ± SD)	1.37 ± 0.4	4 ± 0.92	–	<.001
Education (level)^a^ 12 years ≥ (*n*)	1.17 ± 0.48 2 (8.3%)	1.54 ± 0.97 2 (15.4%)	1.21 ± 0.1 2 (8.3%)	.582
Smokers	3 (12.5%)	2 (13.4%)	4 (16.7%)	.963
Presence of physical disease	20 (83.3%)	11 (84.6%)	20 (83.3%)	.994
Age at onset (years, mean ± SD)	74 ± 5.7	74.3 ± 4.3	–	.817
Using ACE‐I	22 (91.7%)	9 (69.2)	–	.193
Using antidepressant	6 (25%)	5 (38.5%)	–	.632
Using antipsychotic	3 (12.5%)	3 (23.1%)	–	.714
MMSE (mean ± SD)	18.1 ± 2.8 Range = 13–22	11.9 ± 2.1 Range = 8–15	27.3 ± 1.3 Range = 26–30	**<.001** ^b^ (Mild AD‐CN **< .001;** mod. AD‐CN **< .001;** mild‐mod. AD **< .001)**
GDS [median (min–max)]	5 (2–9)	4 (1–6)	3 (1–7)	**.012** ^b^ (Mild AD‐CN **.006;** mod. AD‐CN .131; mild‐mod. AD .32)
FAB [median (min–max)]	11.5 (7–15)	6 (5–8)	15 (13–17)	**<.001** ^b^ (Mild AD‐CN **< .001;** mod. AD‐CN **< .001;** mild‐mod. AD **.005)**

*Note*: Those in bold indicate statistically significant results.

Abbreviations: ACE‐I, acetylcholinesterase inhibitors; AD, Alzheimer's disease; CDR, clinical dementia rating; CN, cognitively normal; FAB, frontal assessment battery; GDS, geriatric depression scale; MMSE, mini‐mental state examination; SD, standard deviation.

^a^ Turkish educational system is categorized into levels from 1 = 5 years of primary education to 5 = academic schooling.

^b^ Pairwise comparison was performed.

As expected, all groups significantly differed in their MMSE scores (*F* = 37.2, all *p* < .001). Regarding the FAB scores, a significant difference was found (*F* = 141.54) between NCs and patients with mild AD (*p* < .001), NCs and patients with moderate AD (*p* < .001), and patients with mild AD and moderate AD (*p* < .001). In addition, significant differences were found between patients with mild and moderate AD in five of the six‐item of the FAB (all *p* < .05), except for conceptualization (*p* > .05). A statistically significant difference was observed in the GDS scores between patients with mild AD and those with NCs (*p* = .006), but not in patients with moderate AD (*p* = .54).

A comparison of the neuropsychological test results of the patients with mild and moderate AD is shown in Table 2. CDR‐SB scores were significantly higher in patients with moderate AD (*t* = 13.24, *p* < .001). Both the Katz ADL and IADL scores were significantly lower in patients with moderate AD (*t* = 8.82 and 5.72, respectively; both *p* < .001). When the NPI‐C symptom domains were compared between the two groups, differences were found in the sleep disorders (*U* = 75.0, *p* = .009) and irritability domains (*U* = 94.5, *p* = .049).

**TABLE 2:** Neuropsychiatric features and functionality of patients with Alzheimer's disease (AD).

Scales	Mild AD (CDR 1)	Moderate AD (CDR 2)	*p*
	(*n* = 24)	(*n* = 13)	
CDR‐SB [median (min–max)]	5.5 (4–8)	11 (8–12)	**<.001**
Katz ADL [median (min–max)]	6 (5–6)	3 (1–6)	**<.001**
IADL (mean ± SD)	5.2 ± 1.1	2.8 ± 1.5	**<.001**
NPI‐C			
Delusions [median (min–max)]	–	0 (0–12)	.058
Hallucinations [median (min–max)]	0 (0–2)	0 (0–2)	.987
Agitation [median (min–max)]	0 (0–9)	5 (0–17)	.077
Aggression [median (min–max)]	0 (0–5)	2 (0–12)	.095
Dysphoria [median (min–max)]	3 (0–13)	0 (0–13)	.089
Anxiety [median (min–max)]	2 (0–18)	0 (0–24)	.888
Elation/euphoria [median (min–max)]	–	0 (0–4)	.718
Apathy/indifference [median (min–max)]	12 (0–21)	0 (0–32)	.962
Irritability/lability [median (min–max)]	0 (0–18)	6 (0–18)	**.049**
Disinhibition [median (min–max)]	0 (0–3)	0 (0–11)	.276
Aberrant motor behavior [median (min–max)]	0 (0–10)	0 (0–12)	.604
Sleep disorders [median (min–max)]	2.5 (0–14)	6 (0–14)	**.009**
Appetite and eating disorders [median (min–max)]	0 (0–4)	0 (0–6)	.985

*Note*: Those in bold indicate statistically significant results.

Abbreviations: CDR‐SB, clinical dementia rating scale‐sum of boxes; IADL, Lawton instrumental activities of daily living; Katz ADL, Katz index of independence in activities of daily living; NPI‐C, neuropsychiatric inventory‐clinician rating scale.

Looking at all AD patients together (*n* = 37), MMSE correlated positively with FAB (*r* = .71, *p* < .001) and GDS (*r* = .35, *p* = .032). Delusions (*r* = −.38, *p* = .021), agitation (*r* = −.39, *p* = .019), irritability/lability (*r* = −.37, *p* = .024), and sleep disorders (*r* = −.37, *p* = .023) domains of the NPI‐C scale were also found to be associated with MMSE in patients.

### 3.2 Facial emotional recognition tasks

**FIGURE 1:**
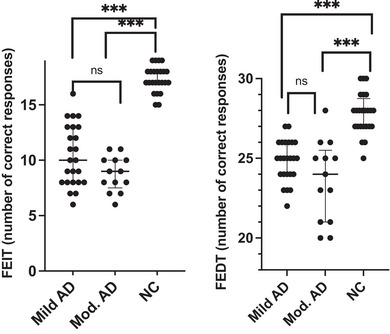
Differences across groups in the facial emotion identification test (FEIT) and facial emotion discrimination test (FEDT).

Figure 1 shows the differences in the abilities of the facial emotional recognition tasks. Regarding FEIT, patients with mild and moderate AD were found to correctly recognize facial emotions at a significantly lower rate than the healthy controls (both *p* < .001). However, no significant difference was observed in the FEIT total score between the patients with mild and moderate AD (*p* > .05). Moreover, Quade's ANCOVA also indicated that FEIT total scores were not still significantly different between patients with mild and moderate AD when adjusted for age, sex, education level, and total duration of illness (*p* > .05). However, after additional adjustment for symptoms of depression (GDS score), the results changed from nonsignificant to significant for FEIT scores in AD patients (*F* = 24.75, *p* = .06).

In terms of facial emotion discrimination test (FEDT), similar to facial emotion identification, both groups of patients with AD were able to discriminate fewer emotions than healthy controls (both *p* < .001). Similar to the FEIT total score, there was no difference in FEDT scores between patients with mild and moderate AD (*p* > .05). Nevertheless, when adjusted for age, sex, education level, and total duration of illness, no significant differences were found between patients with mild and moderate AD regarding FEDT scores (*p* > .05). After additional adjustment for depression symptoms (GDS score), the results remained nonsignificant for the FEDT scores in patients with AD (*p* = .1).

When we conducted a correlation analysis between the NPI‐C with FEIT and FEDT scores of all patients with AD (*n* = 37), none of the domains showed a statistically significant correlation (all *p* > .05). Moreover, all patients had an inverse association between age and FEIT (*r* = −.47, *p* = .004), but not with FEDT (*p* = .18). There were no sex‐related differences in the FEDT and FEIT scores of all the patients (*p* < .05).

In all AD patients, there was a weak or moderate correlation between FEIT score and FAB total and frontal inhibitory control (*r* = .35, *p* = .033; *r* = .42, *p* = .009, respectively), whereas there was a moderate correlation with programming (*r* = .46, *p* = .004). Moreover, weak correlations were found between basic activities of daily living (Katz ADL) and FEIT score (*r* = .33, *p* = .049) and between IADL and FEDT score (*r* = .36, *p* = .036) in all AD patients. Correlation analyses of all patients are presented in the Supporting Information section.

Linear regression models were used to examine the relationships between FEIT, FEDT, and the variables. The models contained three independent variables (age, FAB score, and GDS score). Linear regression analysis indicated that the FEIT total score was significantly related to age (*p* = .004) and FAB score (*p* = .014). FEDT score was not significantly related to any variables (all *p* > .05). Regression models of the factors related to the FEIT total and FEDT scores are presented in Table 3.

**TABLE 3:** Regression models of factors predicting facial emotion identification test (FEIT) and facial emotion discrimination test (FEDT) total scores of patients with Alzheimer's disease (AD).

Mild and moderate AD (*n* = 33)						
	Univariate			Multivariate		
	Beta	*p*‐Value	95% CI	Beta	*p*‐Value	95% CI
**FEIT**						
Age	−.467	**.004**	−.342 to −.073	−.393	**.019**	−.318 to −.03
FAB	.400	**.014**	.071 to .588	.281	.09	−.038 to .501
GDS	.016	.924	−.529 to .581	.029	.856	−.472 to .565
**FEDT**						
Age	−.224	.182	−.187 to .037	−.087	.623	−.148 to .09
FAB	.281	.092	−.03 to .379	.313	.086	−.029 to .417
GDS	−.177	.296	−.627 to .196	−.234	.185	−.715 to .144

*Note*: Those in bold indicate statistically significant results.

Abbreviations: FAB, frontal assessment battery; GDS, geriatric depression scale.

Analysis of the moderation model assessed whether the depression score moderated the relationship between FAB and FEIT or FEDT, after controlling for the effects of covariates (age, sex, total duration of illness, and education level). Depression score was identified as a moderator variable, weakening the preexisting relationship between FAB and FEDT (*b* = −2.018, *t* = −2.635, *p* = .013). Additionally, the interaction term was found to be *R* ^2^ = .38, and when the depression risk score was added as a moderating variable, its contribution to the model was 11.4%. The moderating effect of depression score was found to be more evident, especially when the GDS score was +1 SD (*p* = .05). However, it was found that depression scores did not seem to be a mediating variable in the relationship between FAB and FEIT (*p* = .693).

## 4 DISCUSSION

This study demonstrated a decrease in FER ability in patients with AD compared to CN. However, there was no difference in the FER ability between patients with mild and moderate AD. The FER ability of patients with mild and moderate AD was not significantly affected by confounding factors such as age, sex, education level, and duration of illness. Although FER performance did not differ between the two AD groups, facial emotional identification and discrimination did show a relationship with executive functions and depressive symptoms severity (even though it does meet the diagnosis of depressive disorder). In addition, the FER deficit was not associated with global cognition or neuropsychiatric symptoms.

There is no consensus on how FER is affected in AD, which is the most common type of dementia (Bora et al., 2016). A systematic review of 22 studies published in 2019 stated that the results were ambiguous and showed no consistent findings regarding FER impairment in AD (Torres Mendonça De Melo Fádel et al., 2019). Another review published in 2021 emphasized that impairment in recognition may be a vital sign that facilitates the diagnosis and early treatment of neurodegenerative diseases, such as FTD and AD (González‐Alcaide et al., 2021). In our study, FER capacity was significantly impaired in patients with AD. Few studies have included patients with mild and moderate AD, emphasizing the relationship between disease progression and FER deficits (Bertoux et al., 2015; Torres et al., 2015). The only study comparing FER ability in patients with mild AD and moderate AD found that patients with moderate AD were particularly impaired in their ability to comprehend facial emotions and the expected emotional state in a given situation (Dourado et al., 2019). The present findings point to difficulties in FER throughout the AD stages.

An association between worse executive functions and FER deficits has been reported in schizophrenia (Yang et al., 2015), bipolar disorder (David et al., 2014), borderline personality disorder (Williams et al., 2015), alcohol use disorder (Bora & Zorlu, 2017), and Parkinson's disease (Péron et al., 2012), and this issue has been less evaluated in AD. Studies have reported a relationship between poor performance in executive functions and FER deficit (Phillips et al., 2010), and studies reported no relationship (Sheardova et al., 2014). Executive functions appear to play a role in the regulation of social cognition. Recognition of short‐term displayed emotional expressions may require executive processes such as mental speed, cognitive flexibility, and inhibitory control to suppress one's perspective and to focus attention on relevant features so that all relevant information can be processed promptly (David et al., 2014). In line with the literature, executive function and FER ability may be impaired by prefrontal dysfunction (Adolphs, 2002). In addition to all these, as FAB is also used to assess not only executive function but also frontal lobe functions, including attention, initiation, disinhibition, monitoring, language, and emotion control, the positive relationship between FAB and FEDT/FEIT scores needs to be clarified with new studies.

The severity of depressive symptoms negatively moderated the significant relationship between executive functions and FER ability. As is known, depressive symptoms are common in AD patients (Lyketsos et al., 2002). Meta‐analyses suggest a FER deficit in major depression (Demenescu et al., 2010; Krause et al., 2021). Studies have shown that depression is associated with abnormalities in emotional stimuli's perception, response, and memory storage. Individuals with depression have a negative bias in interpreting facial expressions and a memory bias in which negative information is recalled more (Hale et al., 1998; Leppänen, 2006; Raes et al., 2006). In a study of patients with AD, the presence of depression was found to have a significant negative effect on the recognition of basic emotions and neutral expressions (Weiss et al., 2008). Although we did not include patients with clinical depressive symptoms in our study, we found that subclinical depressive symptoms also negatively affected FER capacity. Even subsyndromal depressive symptoms may significantly affect emotion recognition and may require intervention to improve social cognition in patients with AD.

One of the issues on which there is no consensus in emotion recognition studies in AD is whether the decrease in FER capacity is a specific problem or a reflection of the decrease in global cognitive status. Unlike our results, some studies found a relationship between the decline in emotion recognition skills and the decline in MMSE score (Hargrave et al., 2002; Phillips et al., 2010, Sheardova et al., 2014; Torres et al., 2015; Weiss et al., 2008). In contrast, some studies reported no such strong relationship in AD, similar to our results (Kohler et al., 2005; Lavenu et al., 1999; Roudier et al., 1998; Shimokawa et al., 2000; Taler et al., 2008). Although FER can be considered a noncognitive ability in its own right, cognitive impairments that occur even in the early stages of dementia can interfere with this ability (Torres et al., 2015). Another critical point is that in patients with AD, short‐term visual memory skills are required when deciding whether the previous picture is the same or different from the last picture or which emotion it is. Short‐term visual memory function is also impaired in these patients (Fernández et al., 2018; Norton et al., 2020). Whether the short‐term visual memory impairment is related to emotion recognition or discrimination impairment should be clarified. Longitudinal studies using a more comprehensive test battery are needed to understand better the relationship between emotion recognition and cognitive status in dementia.

There are few studies examining the relationship between neuropsychiatric symptoms and FER ability. In some of these studies, no relationship was found between neuropsychiatric symptoms and FER deficit (Martinez et al., 2018; Torres et al., 2015), whereas a relationship was found in one study (Dourado et al., 2019). We did not find a relationship between any domain of NPI‐C and FER deficit in patients with AD. Our results support that FER ability is not directly linked with neuropsychiatric symptoms. Nevertheless, more research examining the relationship between FER ability and specific neuropsychiatric symptoms is needed as there are few studies on this subject.

Several limitations of our study should be considered. First, this study has a small sample size, and it may not be possible to generalize the results to all AD patients. Second, cognitive impairment was evaluated using MMSE and CDR scores alone. It is unknown whether implementing a more comprehensive neuropsychological battery for cognitive impairment would change the results. Third, patients were under medications, and the effect of anti‐dementia treatments on FER ability is unclear. Fourth, the presence of prosopagnosia in patients with AD was assessed only by history and neurological examination, and no objective test was applied. Finally, the fact that the study was cross‐sectional rather than longitudinal requires caution when interpreting the results (González‐Alcaide et al., 2021; Heilman, 2021).

Our study has some strengths that should be highlighted. We excluded comorbidities of vascular dementia and major depressive disorder in the patients included in the study by using scales, thus eliminating the effect of these factors on the results and obtaining a more homogeneous sample. Nevertheless, we obtained the striking result that even subsyndromal depressive symptoms affect FER ability. We used the CDR scale instead of the MMSE total score to make a more accurate staging of dementia in the mild or moderate AD group. We matched all groups’ age and education levels perfectly, which may have contributed to the results. The scope of the study was expanded by evaluating the FER capacities of the patients with their depression, neuropsychiatric symptoms, executive functions, and activities of daily living and examining their relationship with each other.

In conclusion, the present study showed that FER capacity decreased in both mild and moderate AD compared to healthy controls. The critical point in FER deficit is the presence of dementia, not the dementia stage. The FER deficits in AD go beyond a global cognitive deficit, are related to executive functions, and are exacerbated by depressive symptoms. It may be appropriate for clinicians to be aware of the FER deficit in AD patients and to be directed to social cognition training, considering that it has the potential to improve impaired emotion recognition and social functioning in individuals with FER deficit, which may contribute to reducing caregiver burden (Schoeneman Patel et al., 2022). To better identify deficits in emotional processing in AD, we believe that longitudinal studies with larger sample sizes that examine FER, as well as other aspects of emotional processing, are needed, where potential neural mechanisms can be identified and the efficacy of treatments evaluated.

## AUTHOR CONTRIBUTIONS

**İsmail Buçgün**: Conceptualization; investigation; writing—original draft; data curation; methodology; project administration. **Şükrü Alperen Korkmaz**: Software; formal analysis; data curation; supervision; writing—review and editing; visualization; validation; resources. **Demet Güleç Öyekçin**: Project administration; resources; methodology; validation; funding acquisition; conceptualization; supervision; writing—review and editing.

## CONFLICT OF INTEREST STATEMENT

The authors declare no conflicts of interest for this article.

## FUNDING INFORMATION

This research received no external funding.

### PEER REVIEW

The peer review history for this article is available at https://publons.com/publon/10.1002/brb3.3390.

## PATIENT CONSENT STATEMENT

Healthy subjects and all patients with AD could provide signed, informed consent. The family caregivers also gave informed consent before the interview.

## Supporting information


**Supplementary Table** Correlation analysis results (*r*‐values) of all AD patients (*n* = 37)

## Data Availability

The data presented in this study are available on request from the corresponding author. The data are not publicly available due to ethical and institutional reasons.
