# Supplementary material for: Facial emotion recognition is associated with executive functions and depression scores, but not staging of dementia, in mild‐to‐moderate Alzheimer's disease
Source: Brain Behav. 2024 Jan 24;14(1):e3390. doi: 10.1002/brb3.3390 (PMC10808849; doi:10.1002/brb3.3390)
Supplement: Supplementary file 1 — Supplementary Table Correlation analysis results (r‐values) of all AD patients (n = 37) [file BRB3-14-e3390-s001.docx]

**Supplementary Table.** Correlation analysis results (*r*-values) of all AD patients (n=37)

| Variables | FEIT | FEDT | MMSE | GDS | FAB total | NPI-C delusions | NPI-C hallucinations | NPI-C agitation | NPI-C aggression | NPI-C dysphoria | NPI-C anxiety | NPI-C elation/euphoria | NPI-C apathy | NPI-C irritability/lability | NPI-C disinhibition | NPI-C aberrant motor behaviour | NPI-C sleep disorder |
| --- | --- | --- | --- | --- | --- | --- | --- | --- | --- | --- | --- | --- | --- | --- | --- | --- | --- |
| FEIT | 1 |  |  |  |  |  |  |  |  |  |  |  |  |  |  |  |  |
| FEDT | 0.168 | 1 |  |  |  |  |  |  |  |  |  |  |  |  |  |  |  |
| MMSE | 0.227 | 0.189 | 1 |  |  |  |  |  |  |  |  |  |  |  |  |  |  |
| GDS | -0.177 | -0.188 | 0.354* | 1 |  |  |  |  |  |  |  |  |  |  |  |  |  |
| FAB total | 0.352* | 0.225 | 0.714** | 0.188 | 1 |  |  |  |  |  |  |  |  |  |  |  |  |
| NPI-C delusions | -0.118 | -0.169 | -0.38* | -0.028 | -0.438** | 1 |  |  |  |  |  |  |  |  |  |  |  |
| NPI-C hallucinations | 0.116 | 0.158 | 0.188 | 0.395* | 0.103 | 0.147 | 1 |  |  |  |  |  |  |  |  |  |  |
| NPI-C agitation | 0.219 | -0.012 | -0.39* | 0.032 | -0.294 | 0.448* | 0.353* | 1 |  |  |  |  |  |  |  |  |  |
| NPI-C aggression | 0.167 | 0.073 | -0.206 | -0.227 | -.236 | 0.292 | 0.17 | 0.54** | 1 |  |  |  |  |  |  |  |  |
| NPI-C dysphoria | 0.026 | 0.238 | 0.181 | 0.056 | 0.165 | -0.42* | 0.001 | -0.111 | 0.007 | 1 |  |  |  |  |  |  |  |
| NPI-C anxiety | -0.123 | 0.064 | 0.055 | 0.156 | 0.015 | -0.208 | 0.232 | 0.149 | 0.087 | 0.246 | 1 |  |  |  |  |  |  |
| NPI-C elation/euphoria | -0.134 | 0.04 | -0.094 | -0.081 | -0.267 | -0.066 | -0.049 | 0.126 | 0.206 | 0.17 | 0.083 | 1 |  |  |  |  |  |
| NPI-C apathy | -0.072 | -0.048 | -0.142 | -0.134 | -0.095 | -0.487* | -0.2 | -0.426* | -0.286 | 0.238 | -0.052 | 0.206 | 1 |  |  |  |  |
| NPI-C irritability/lability | 0.263 | 0.115 | -0.37* | -0.115 | -0.165 | 0.43* | 0.112 | 0.742** | 0.787** | -0.094 | 0.145 | 0.138 | -0.427* | 1 |  |  |  |
| NPI-C disinhibition | 0.267 | 0.093 | -0.16 | 0.075 | -0.288 | 0.247 | 0.305 | 0.611** | 0.508** | 0.005 | 0.118 | 0.388* | -0.242 | 0.507** | 1 |  |  |
| NPI-C aberrant motor behaviour | 0.055 | 0.222 | -0.13 | -0.152 | -0.240 | 0.163 | 0.251 | 0.394* | 0.235 | 0.216 | -0.059 | 0.319 | 0.02 | 0.34* | 0.51** | 1 |  |
| NPI-C sleep disorder | -0.124 | 0.108 | -0.372* | 0.035 | -0.403* | 0.501* | 0.228 | 0.566** | 0.284 | 0.025 | 0.296 | 0.12 | -0.379* | 0.412** | 0.492* |  | 1 |

*p < 0.05, **p≤ 0.001.

Note: FEIT, Facial Emotion Identification Test; FEDT, Facial Emotion Discrimination Test; MMSE, Mini-Mental State Examination; FAB, Frontal Assessment Battery; GDS, Geriatric Depression Scale (GDS); NPI-I, Neuropsychiatric Inventory-Clinican Rating Scale.
